# An interpretable ultrasound-based deep learning system for early breast cancer in a Chinese population

**DOI:** 10.1186/s13244-026-02323-3

**Published:** 2026-06-04

**Authors:** Siqi Wu, Shenwen Wang, Di Liang, Jin Shi, Xiao Shang, Liwen Zhang, Yanyu Liu, Xin Su, Yi Wang, Qian Lu, Zheng Li, Zhenfeng Zhao, Xiaohui Ji, Daojuan Li, Yutong He

**Affiliations:** 1 Cancer Institute, The Fourth Hospital of Hebei Medical University, Shijiazhuang, China; 2 School of Information Engineering, Hebei GEO University, Shijiazhuang, China; 3 School of Public Health, Hebei Medical University, Shijiazhuang, China; 4 Department of Ultrasound, The Fourth Hospital of Hebei Medical University, Shijiazhuang, China; 5 Hebei Huiji Technology Development Co., Ltd., Shijiazhuang, China

**Keywords:** Breast neoplasms, Ultrasonography, Deep learning, Early diagnosis, Artificial intelligence

## Abstract

**Objectives:**

Current deep learning models for early breast cancer lack interpretability and multimodal integration, limiting their clinical acceptance. This study aimed to develop and evaluate a deep learning system that automates breast ultrasound evaluation to support early breast cancer detection in clinical assessment.

**Materials and methods:**

We developed BrcaDetect, which integrates ultrasound image-based deep learning predictions, Breast Imaging Reporting and Data System (BI-RADS) assessments, and demographic factors. A total of 24,762 ultrasound images from 3048 women across five hospitals were retrospectively collected. The model was trained and internally validated using 19,340 images from 2399 patients at three tertiary hospitals between January 2017 and December 2020, and externally validated using 5422 images from 649 women at two additional hospitals between January 2021 and August 2023. All lesions were confirmed by biopsy or 3-year follow-up. Model performance and its impact on the diagnostic accuracy of five radiologists were evaluated.

**Results:**

BrcaDetect outperformed image-based deep learning and demographic model, achieving an area under the curve (AUC) of 0.989 (95% confidence interval (CI): 0.979–0.999), 0.851 (95% CI: 0.819–0.884), and 0.826 (95% CI: 0.804–0.848), with corresponding sensitivities of 98.8%, 93.0%, and 71.8%. In the reader study, radiologists assisted by BrcaDetect achieved significantly higher diagnostic accuracy than unassisted reading (0.977 [95% CI: 0.967–0.986] vs. 0.919 [95% CI: 0.900–0.935]; *p* < 0.001).

**Conclusions:**

As an image‑level decision-support model, BrcaDetect was associated with improved radiologists’ performance and interpretability under controlled reading conditions, reducing false positives and demonstrating proof-of-concept for decision support in clinical assessment workflows.

**Key Points:**

- Current deep learning models for early breast cancer lack interpretability and multimodal integration, severely limiting their clinical acceptance in practice.
- In a retrospective study, BrcaDetect outperformed single‑modality models across three cohorts and provided strong interpretability via Grad‑CAM and Shapley values.
- This article addresses the lack of interpretability and multimodal integration in deep learning models for early breast cancer, presenting BrcaDetect: explainable predictions via Grad-CAM and Shapley values may reduce diagnostic uncertainty and support clinical workflow integration as a proof-of-concept.

**Graphical Abstract:**

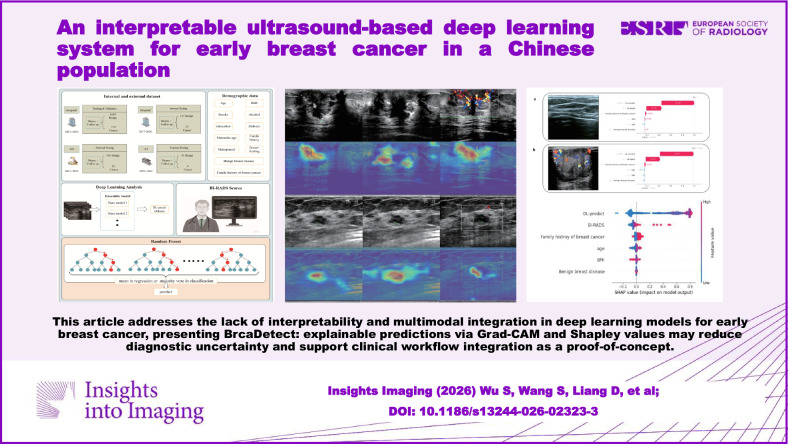

## Introduction

Breast cancer is the most commonly diagnosed malignancy and the leading cause of cancer-related death among women worldwide [1, 2]. Early detection markedly improves survival, underscoring the need for accurate and non-invasive diagnostic tools [3–5]. Although screening mammography remains the cornerstone of population-based screening, its performance is limited by false-positive findings and inter-reader variability, particularly in women with dense breast tissue [6]. Breast ultrasound is therefore widely applied as a radiation-free and cost-effective modality, especially for dense breasts [7]. However, its clinical utility is constrained by relatively low specificity and strong operator dependence, leading to inconsistent interpretation in large-scale screening settings [8, 9].

Deep learning (DL) has shown promise in cancer detection and classification [10]. In breast ultrasound, most DL studies focus on differentiating benign and malignant lesions in diagnostic cohorts, often using single-center datasets and image-only features [11, 12]. Such approaches may not reflect the heterogeneity of population-based screening and frequently lack robust external validation. Moreover, many DL models function as black boxes, limiting clinical interpretability. Readily available clinical and demographic risk factors, such as age, breastfeeding, reproductive history, and family history of breast cancer, are often omitted despite their established relevance [13]. Prior work has also largely focused on B-mode ultrasound images, overlooking complementary information from color Doppler imaging, which can improve breast cancer risk prediction [14, 15].

To address these gaps, we developed BrcaDetect, an interpretable multimodal DL framework for early breast cancer identification. The system integrates DL-based image predictions with Breast Imaging Reporting and Data System (BI-RADS) assessments and key demographic risk factors to improve diagnostic performance while enhancing transparency. Model performance was evaluated against radiologist assessments, and the potential to support clinical decision-making was examined. In addition to breast cancer detection, BrcaDetect provides explanatory outputs by visualizing informative image regions using Gradient-weighted Class Activation Mapping (Grad‑CAM) heatmaps and quantifying the contribution of individual variables using Shapley values. We hypothesized that this multimodal and interpretable approach, as an image-level aid, might improve diagnostic performance and clinical interpretability, supporting the integration of AI-assisted ultrasound analysis into breast cancer screening workflows.

## Methods

### Study design and participants

We retrospectively collected data from patients who underwent ultrasound examinations at three tertiary care hospitals–The First Hospital of Hebei Medical University, The Fourth Hospital of Hebei Medical University, and Hebei Chest Hospital–between January 2017 and December 2020. Disease stage at diagnosis was determined according to the American Joint Committee on Cancer (AJCC) staging system, 7th edition [16]. Early-stage disease was defined as stage I breast cancer. The study protocol was approved by the Human Ethics Committee of the National Cancer Center/Cancer Hospital, Chinese Academy of Medical Sciences and Peking Union Medical College (No. 18-016/1645), and The Fourth Hospital of Hebei Medical University (No. 2025KS111). Written informed consent was obtained from all participants before ultrasound image acquisition.

Malignant lesions were confirmed by histopathological examination of core needle biopsy or surgical specimens. Benign lesions were defined by histopathological confirmation or by imaging stability without evidence of malignancy during a minimum follow-up period of 3 years. Consecutive patients were eligible if they met the following criteria: (1) a clearly identifiable primary breast lesion on ultrasound; (2) pathological confirmation for the target lesion by needle biopsy, surgical specimen, or follow-up records; and (3) availability of ultrasound images acquired in the longest-axis plane of the lesion. Exclusion criteria were: (1) inconclusive pathological or follow-up results; (2) a prior biopsy of the same breast lesion before the ultrasound examination; and (3) inadequate image quality that precluded manual segmentation. The patient recruitment process, detailed inclusion and exclusion criteria are shown in Supplementary Fig. S1. Demographic characteristics and breast cancer-related-risk factors were collected using standardized questionnaires; detailed information is provided in Supplementary Material 1.

Independent validation of the DL system was conducted in consecutive screening cohorts from Shijiazhuang People’s Hospital (January 31, 2021–August 1, 2023) and Xingtai People’s Hospital (January 31, 2021–February 3, 2022). Screening data and outcomes were obtained within the framework of the Cancer Screening Program in Urban China (CanSUPC). Women aged 40 to 74 years without a previous cancer diagnosis or serious health conditions were recruited through telephone calls, face-to-face contact, social media, and community advertisements. Breast cancer risk assessment was performed using a scoring system derived from the Harvard Index score [17]. Women classified as having increased breast cancer risk were advised to undergo ultrasound and mammography (MAM) examinations. Detailed information on the risk assessment score is provided in Supplementary Material 1.

### Image collection

Ultrasound images, including B-mode and color Doppler images, were retrospectively obtained from women with breast lesions at the five participating tertiary care hospitals. All ultrasound examinations and BI-RADS category assignments were performed by radiologists with more than 5 years of experience. Ultrasound systems from multiple vendors were used in this study; detailed information on vendors, systems, and probe specifications is provided in Supplementary Table 1. A harmonized scanning protocol was implemented to reduce inter-center variability. This protocol standardized patient positioning (supine with the ipsilateral arm raised), image acquisition in orthogonal planes, and documentation of key lesion characteristics. Each lesion was examined in at least two perpendicular planes (longitudinal and transverse), with and without caliper measurements. Additional greyscale ultrasound sections showing suspicious malignant features were selectively acquired. For each lesion, images were captured at the maximum diameter. Suspicious cases were confirmed by pathological biopsy, whereas benign lesions were verified by biopsy or by a 3-year follow-up.

### Dataset

**Fig. 1:**
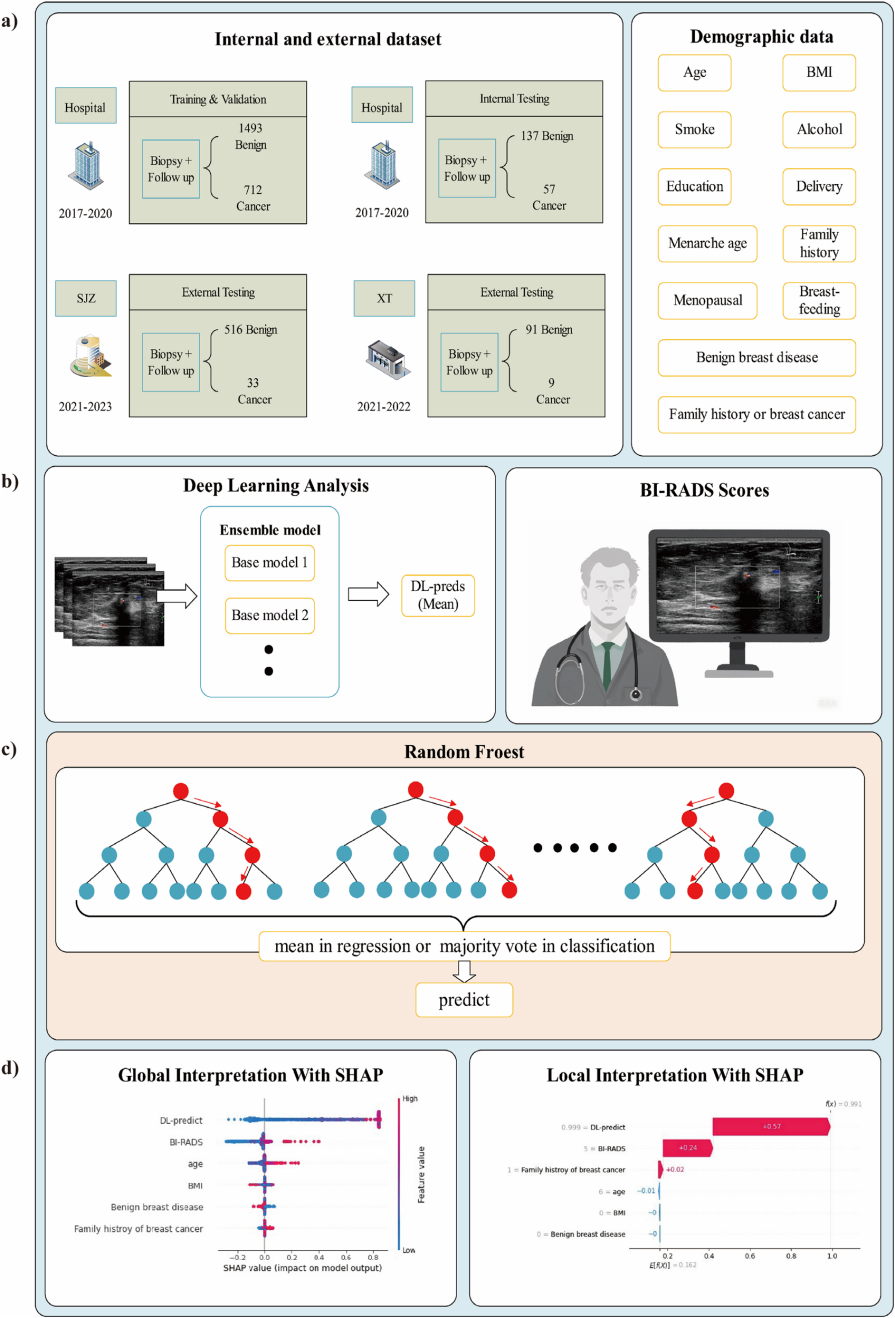
Development of BrcaDetect using image-based deep learning predictions, BI-RADS scores from radiologists, and demographic parameters. **a** Data acquisition; **b** multimodal information; **c** model development; and **d** global and local interpretation of BrcaDetect. BI-RADS, Breast Imaging Reporting and Data System

In this study, ultrasound images were treated as the primary unit of analysis, and BrcaDetect was designed and evaluated as an image-level decision-support system rather than a patient-level diagnostic tool. Ultrasound images were retrospectively collected from women with breast lesions across five hospitals (Fig. 1). To prevent data leakage and maintain dataset independence, data splitting was performed at the participant level rather than the image level. All images from the same woman were assigned to a single dataset only. In addition, images acquired during the same examination session were kept together and were not distributed across different datasets. Data from three hospitals were used for model development and internal evaluation and were randomly divided into training, validation, and internal test datasets at a ratio of 7:2:1. Two additional hospitals were reserved exclusively for external validation. No overlap of participants occurred among the training, validation, internal test, and external test datasets.

### Model development and evaluation

#### Deep learning prediction model

A DL prediction model was developed using ultrasound images alone to detect breast cancer, without the incorporation of additional information. An ensemble comprising eight backbone architectures was implemented, including ResNet34, ResNet50, ResNet101 [18], EfficientNet-b5, EfficientNet-b6 [19], DenseNet121, DenseNet169, and DenseNet201 [20]. All models were first pre-trained on ImageNet [21] and subsequently fine-tuned using the training dataset. Identical training parameters were applied across models. The Adam optimizer was used with a learning rate of 0.0001. Input images were resized to 512 × 512 pixels, with a batch size of 32. To improve generalization during training, data augmentation strategies such as random horizontal flipping, rotation, and color jittering were applied. Models were trained for 100 epochs and evaluated on the validation dataset after each epoch. Model weights corresponding to the highest area under the curve (AUC) on the validation dataset were selected. Final DL scores were generated by averaging the output probabilities from the eight models.

### BrcaDetect and demographic model

Random forest (RF) algorithms were used to construct both BrcaDetect and the demographic model. BrcaDetect was trained on six-dimensional feature vectors combining four demographic factors (age, BMI, history of benign breast disease, and family history of breast cancer), BI-RADS assessment categories, and DL‑derived scores (Fig. 1). Although BI-RADS represents an interpretive assessment provided by radiologists rather than an independent quantitative feature, it was deliberately included to mirror routine clinical practice, in which radiologist judgment and algorithmic outputs are considered together. This multimodal feature integration reflects a balance between strict feature independence and clinical applicability and was adopted to assess whether combining expert-derived assessments with automated image-based scores could improve early breast cancer detection in real-world settings.

The demographic model was built using only the four demographic factors that showed statistical significance, resulting in four-dimensional input vectors (Supplementary Table 2). For RF training, the number of estimators (*N*) was predefined. Bootstrapping was applied to generate simulated datasets for each estimator by randomly resampling the training data with replacement 1000 times. Decision trees were constructed using these simulated datasets, yielding an ensemble of N trees with heterogeneous structures. Final predictions were obtained by aggregating outputs from all decision trees using majority voting.

### Interpretation of BrcaDetect

**Fig. 2:**
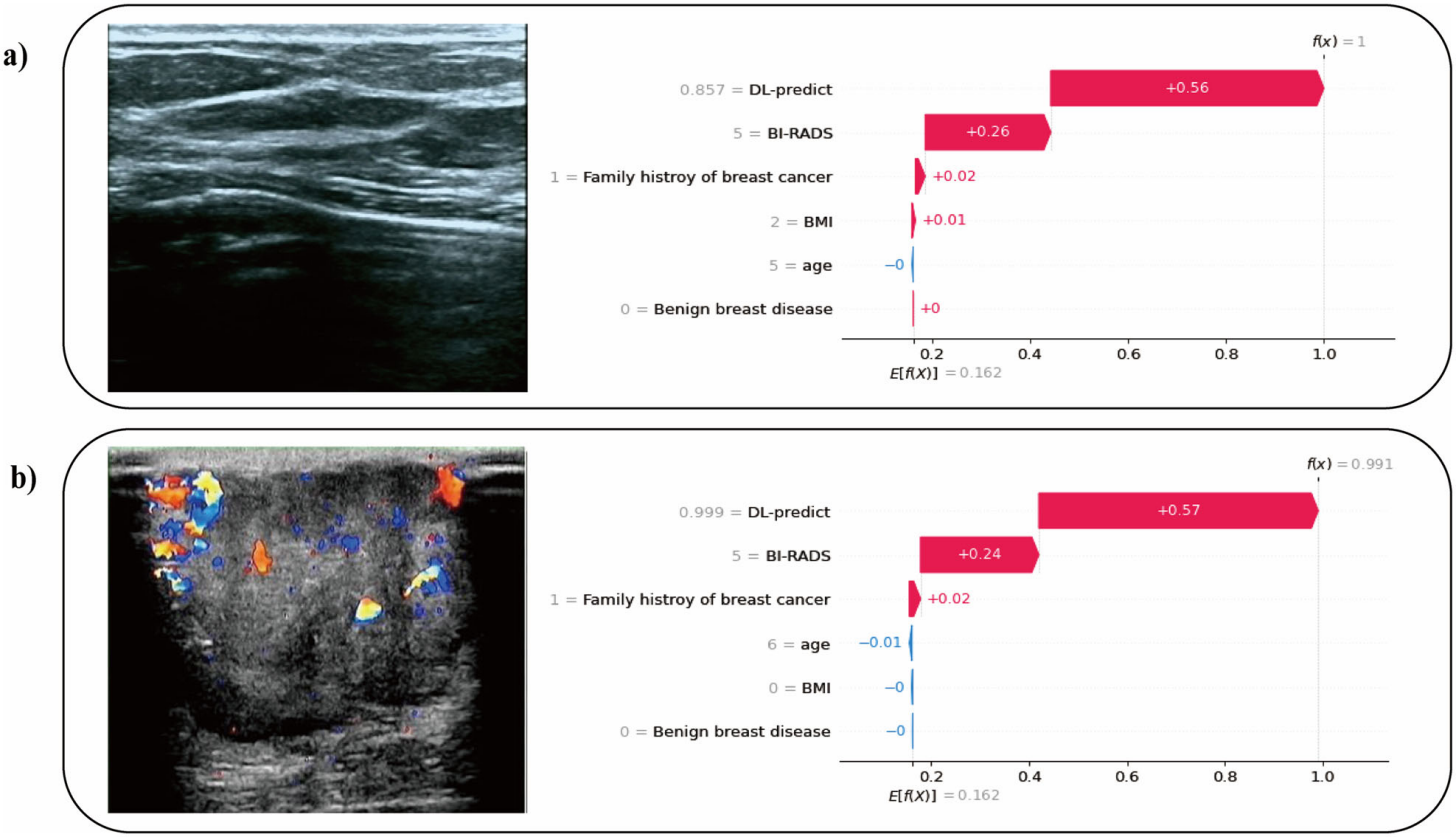
Local Shapley values for the interpretation of BrcaDetect. The horizontal ordinate represents the decision score. *f* is the function of BrcaDetect, *f*(*x*) is the final decision probability for input *x*, and *E*[*f*(*X*)] is the expected value (mean value) for BrcaDetect’s decision probabilities of all training samples (which is 0.162). For sample **a**, the DL model prediction values, BI-RADS score, and family history of breast cancer were 0.857 (prediction probability), 5, and yes, giving +0.56, +0.26, and +0.02 contributions supporting the decision to apply a malignant label, respectively. These contributions were then added to the expected value to obtain the final decision probability of 1.00. For sample **b**, the DL model prediction values, BI-RADS score, and family history of breast cancer were 0.999 (prediction probability), 5, and yes, giving +0.57, +0.24, and +0.02 contributions supporting the decision to apply a malignant label. In addition, the age was 66 years, giving a −0.01 negative impact against the decision to apply a malignant label. These contributions were then added to the expected value to obtain the final decision probability of 0.991

Grad‑CAM heatmaps and Shapley values were used to improve the interpretability of BrcaDetect. Grad‑CAM heatmaps were applied to provide visual insight into image regions contributing to malignancy predictions [22]. In parallel, the relative contribution of each input feature was quantified using Shapley values. Instance-level Shapley values were calculated for each factor to explain individual predictions (Fig. 2). Global Shapley values were obtained by averaging local Shapley values across all samples, representing the mean effect of each factor on model output.

### Definition of hotspots

To further support interpretability, Grad‑CAM was used to visualize image regions that contributed most strongly to model predictions [23]. Grad-CAM heatmaps were normalized to the [0,1] range to reflect relative activation intensity. Regions with higher activation values were qualitatively defined as “hotspots”, corresponding to discriminative image features highlighted by the model and indicating lesion-related areas.

### Reader study

To compare the performance of BrcaDetect with that of radiologists, a reader study was conducted using cases drawn from the internal and external test datasets, with the participation of five radiologists (R1 to R5). Radiologists R2 and R5 had up to 10 years of clinical experience, whereas R1, R3, and R4 had approximately 5 years of experience. None of the participating radiologists were involved in the development of the BrcaDetect model. Before formal evaluation, all radiologists completed a standardized training session using a separate dataset of 100 breast cases (50 benign and 50 malignant) that were excluded from the test datasets. This session was followed by a consensus discussion led by two senior breast imaging specialists to harmonize assessment criteria and interpretation strategies.

During the formal evaluation, radiologists independently interpreted 200 anonymized cases randomly selected from three test datasets. All readers were blinded to clinical and pathological information. Case order was randomized, and image interpretation was performed under standardized picture archiving and communication system (PACS) conditions, with uniform monitors, lighting, and resolution. A 2-week washout period was applied between training and formal evaluation to minimize recall bias.

To assess the effect of model assistance, the same cases were re-evaluated 2 weeks after the initial reading. During the assisted session, each radiologist first recorded an initial BI-RADS assessment without access to the model output. The model-predicted malignancy probability was then disclosed, and any subsequent change in assessment was documented. A reference diagnosis generated by the DL model was available for comparison. When discrepancies occurred, radiologists could retain their original assessment or adopt the model-suggested diagnosis. The final assessment in the assisted session reflected the integration of radiologist interpretation and model information.

### Statistical analysis

Categorical and continuous variables were summarized as frequencies (percentages) and means ± standard deviations, respectively. Analysis of variance (ANOVA) was used for intergroup comparisons. Model performance was assessed using AUC, sensitivity, specificity, accuracy, positive predictive value (PPV), and negative predictive value (NPV), with corresponding 95% confidence intervals (CIs). AUCs were compared using the DeLong test. Interobserver agreement was evaluated using Cohen’s kappa statistics [24]. All statistical tests were two-tailed, and *p* < 0.05 was considered statistically significant. Statistical analyses were performed using R version 4.2.1 and Python version 3.9.

## Results

### Baseline information

As shown in Fig. 1 and Table 1, this study included 24,762 qualified B-mode and color Doppler ultrasound images from 3048 women (mean age, 55.21 ± 8.40 years; range, 40–74 years). Of these, 19,340 clinical diagnostic ultrasound images from 2399 women collected at three hospitals were assigned to the training, validation, and internal test datasets. The remaining 5422 ultrasound images from screening cohorts from 649 women, obtained from two independent hospitals, constituted the external test datasets.

**Table 1:** Baseline characteristics of the participants

Characteristic	Development datasets		External datasets	
	Train and validation (*n* = 2205)	Internal test (*n* = 194)	SJZ dataset (*n* = 549)	XT dataset (*n* = 100)
Age (mean (SD), years)	55.27 (8.34)	56.98 (8.58)	53.32 (8.22)	55.38 (6.76)
BMI (mean (SD), kg/m^2^)	24.37 (3.13)	24.42 (3.11)	24.92 (3.15)	24.63 (2.64)
Age group, years				
40–44	209 (9.5)	18 (9.3)	80 (14.6)	0 (0.0)
45–49	444 (20.1)	26 (13.4)	138 (25.1)	21 (21.0)
50–54	438 (19.9)	31 (16.0)	98 (17.9)	27 (27.0)
55–59	373 (16.9)	36 (18.6)	99 (18.0)	26 (26.0)
60–64	416 (18.9)	46 (23.7)	72 (13.1)	15 (15.0)
65–69	196 (8.9)	21 (10.8)	42 (7.7)	7 (7.0)
70–74	129 (5.9)	16 (8.2)	20 (3.6)	4 (4.0)
BMI (kg/m^2^)				
18.5–23.9	1072 (48.6)	89 (45.9)	211 (38.4)	44 (44.0)
< 18.5	9 (0.4)	1 (0.5)	7 (1.3)	2 (2.0)
24.0–27.9	838 (38.0)	79 (40.7)	238 (43.4)	42 (42.0)
≥ 28.0	286 (13.0)	25 (12.9)	93 (16.9)	12 (12.0)
Smoking status				
Never	2052 (93.1)	185 (95.4)	514 (93.6)	98 (98.0)
Current	128 (5.8)	8 (4.1)	27 (4.9)	1 (1.0)
Ever	25 (1.1)	1 (0.5)	8 (1.5)	1 (1.0)
Alcohol consumption				
Never	1955 (88.7)	175 (90.2)	462 (84.2)	88 (88.0)
Current	222 (10.1)	13 (6.7)	72 (13.1)	12 (12.0)
Ever	28 (1.3)	6 (3.1)	15 (2.7)	0 (0.0)
Age of menarche (years)				
≥ 13	1853 (84.0)	174 (89.7)	453 (82.5)	85 (85.0)
< 13	352 (16.0)	20 (10.3)	96 (17.5)	15 (15.0)
Menopausal status				
Premenopausal	829 (37.6)	62 (32.0)	244 (44.4)	41 (41.0)
Postmenopausal	1376 (62.4)	132 (68.0)	305 (55.6)	59 (59.0)
Delivery history				
No	138 (6.3)	9 (4.6)	29 (5.3)	5 (5.0)
Yes	2067 (93.7)	185 (95.4)	520 (94.7)	95 (95.0)
Education level^a^				
Low	235 (10.7)	20 (10.3)	40 (7.3)	6 (6.0)
Medium	1502 (68.1)	139 (71.6)	372 (67.8)	74 (74.0)
High	468 (21.2)	35 (18.0)	137 (25.0)	20 (20.0)
Breastfeeding				
No	220 (10.0)	24 (12.4)	44 (8.0)	10 (10.0)
Yes	1985 (90.0)	170 (87.6)	505 (92.0)	90 (90.0)
Personal history of benign breast disease				
No	1464 (66.4)	120 (61.9)	211 (38.4)	72 (72.0)
Yes	741 (33.6)	74 (38.1)	338 (61.6)	28 (28.0)
Family history of breast cancer				
No	1462 (66.3)	134 (69.1)	216 (39.3)	60 (60.0)
Yes	743 (33.7)	60 (30.9)	333 (60.7)	40 (40.0)

Data are presented as mean (standard deviation) for continuous variables and number (percentage) for categorical variables

The development datasets consisted of participants from three hospitals and included the training, validation, and internal test cohorts. The SJZ and XT datasets represent two independent external screening cohorts collected from Shijiazhuang People’s Hospital and Xingtai People’s Hospital, respectively. All characteristics were analyzed at the participant level

*BMI* body mass index, *SD* standard deviation

^a^ Education level was categorized as low (primary school or below), medium (junior or senior high school), and high (college or above)

All cases were assessed by experienced radiologists according to the BI-RADS. The reference standard was established by pathological confirmation or by a 3-year follow-up. Detailed distributions of participants and images across datasets, as well as the numbers of benign and malignant cases, are provided in Table 1 and Supplementary Fig. S1.

### Performance of the demographic model

The demographic model achieved an AUC of 0.794 (95% CI: 0.766–0.822) on the internal test dataset, with a sensitivity of 86.2% and a specificity of 65.6% (Table 2). Performance declined in the external SJZ and XT datasets. AUCs ranged from 0.674 (95% CI: 0.615–0.734) to 0.698 (95% CI: 0.670–0.726), sensitivities ranged from 59.2% to 68.1%, and specificities ranged from 70.1% to 76.7%. The model incorporated four significant predictors: age, BMI, history of benign breast disease, and family history of breast cancer. These predictors were identified through univariate and multivariate logistic regression analyses (Supplementary Table S2).

**Table 2:** The diagnostic performance of the image-based deep learning model, demographic model, and the BrcaDetect

	Demographic model	Image-based DL predictions	BrcaDetect
Internal dataset			
AUC [95% CI]	0.794 [0.766–0.822]	0.953 [0.934–0.972]	0.989 [0.979–0.999]
*p*-value	2.2 × 10^−16^	0.0003077	Reference
Sensitivity (%)	0.862 [0.817–0.903]	0.869 [0.828–0.910]	0.989 [0.974–1.000]
Specificity (%)	0.656 [0.631–0.680]	0.996 [0.993–0.999]	0.982 [0.975–0.988]
Accuracy (%)	0.689 [0.668–0.711]	0.976 [0.969–0.982]	0.983 [0.977–0.988]
PPV (%)	0.328 [0.310–0.348]	0.979 [0.959–0.996]	0.914 [0.884–0.943]
NPV (%)	0.961 [0.949–0.972]	0.975 [0.968–0.983]	0.998 [0.995–1.000]
FPR (%)	0.344 [0.319–0.370]	0.004 [0.001–0.008]	0.018 [0.012–0.027]
SJZ dataset			
AUC [95% CI]	0.698 [0.670–0.726]	0.809 [0.784–0.833]	0.826 [0.804–0.848]
*p*-value	2.2 × 10^−16^	0.0004205	Reference
Sensitivity (%)	0.681 [0.634–0.723]	0.674 [0.632–0.716]	0.718 [0.676–0.758]
Specificity (%)	0.701 [0.686–0.716]	0.840 [0.828–0.851]	0.868 [0.857–0.879]
Accuracy (%)	0.699 [0.684–0.714]	0.820 [0.809–0.832]	0.850 [0.839–0.861]
PPV (%)	0.232 [0.217–0.246]	0.358 [0.335–0.379]	0.419 [0.394–0.444]
NPV (%)	0.943 [0.935–0.950]	0.951 [0.945–0.957]	0.959 [0.953–0.965]
FPR (%)	0.299 [0.284–0.315]	0.160 [0.148–0.173]	0.132 [0.121–0.144]
XT dataset			
AUC [95% CI]	0.674 [0.615–0.734]	0.803 [0.743–0.864]	0.851 [0.819–0.884]
*p*-value	5.769 × 10^−9^	0.07908	Reference
Sensitivity (%)	0.592 [0.479–0.691]	0.620 [0.497–0.732]	0.930 [0.843–0.977]
Specificity (%)	0.767 [0.747–0.788]	0.897 [0.881–0.912]	0.803 [0.782–0.823]
Accuracy (%)	0.759 [0.738–0.780]	0.885 [0.869–0.900]	0.809 [0.789–0.828]
PPV (%)	0.108 [0.087–0.128]	0.223 [0.185–0.266]	0.184 [0.165–0.202]
NPV (%)	0.975 [0.968–0.981]	0.980 [0.975–0.986]	0.996 [0.992–0.999]
FPR (%)	0.233 [0.211–0.255]	0.103 [0.088–0.119]	0.197 [0.177–0.218]

### Performance of DL predictions

Across the eight DL models, AUCs ranged from 0.941 to 0.962 on the internal test dataset and decreased on the external datasets (SJZ: 0.705–0.810; XT: 0.626–0.845) (Supplementary Table S3). The ensemble model achieved the best internal performance, with an AUC of 0.953 and corresponding sensitivity and specificity of 86.9% and 99.6%, respectively (Table 2). On the external datasets, AUCs ranged from 0.803 to 0.809, with sensitivities of 62.0–67.4% and specificities of 84.0–89.7%.

### Performance of BI-RADS

After training, the five radiologists showed strong diagnostic performance in classifying breast lesions. For AUC analysis, BI-RADS categories were normalized to a scale from 0 to 1. The mean AUC was 0.919 on the internal test dataset and 0.751 and 0.670 on the two external datasets (Supplementary Table S4). On the internal dataset, mean sensitivity and specificity were 87.5% and 82.8%, respectively. On the external datasets, sensitivity ranged from 55.2% to 70.5%, and specificity ranged from 71.1% to 73.5%.

### Performance of the BrcaDetect

As shown in Supplementary Fig. S2, BrcaDetect demonstrated superior performance across all datasets. In the internal test dataset, it achieved an AUC of 0.989 (95% CI: 0.979–0.999), exceeding both the demographic model (AUC, 0.794; *p* < 0.001) and the DL ensemble model (AUC, 0.953; *p* < 0.001) (Table 2). Performance remained stable in the external SJZ and XT datasets, with AUCs of 0.826 (95% CI: 0.804–0.848) and 0.851 (95% CI: 0.819–0.884), respectively, consistently higher than those of the demographic models (both *p* < 0.001) and higher than or comparable to those of the DL models (*p* < 0.001 and *p* = 0.080).

**Fig. 3:**
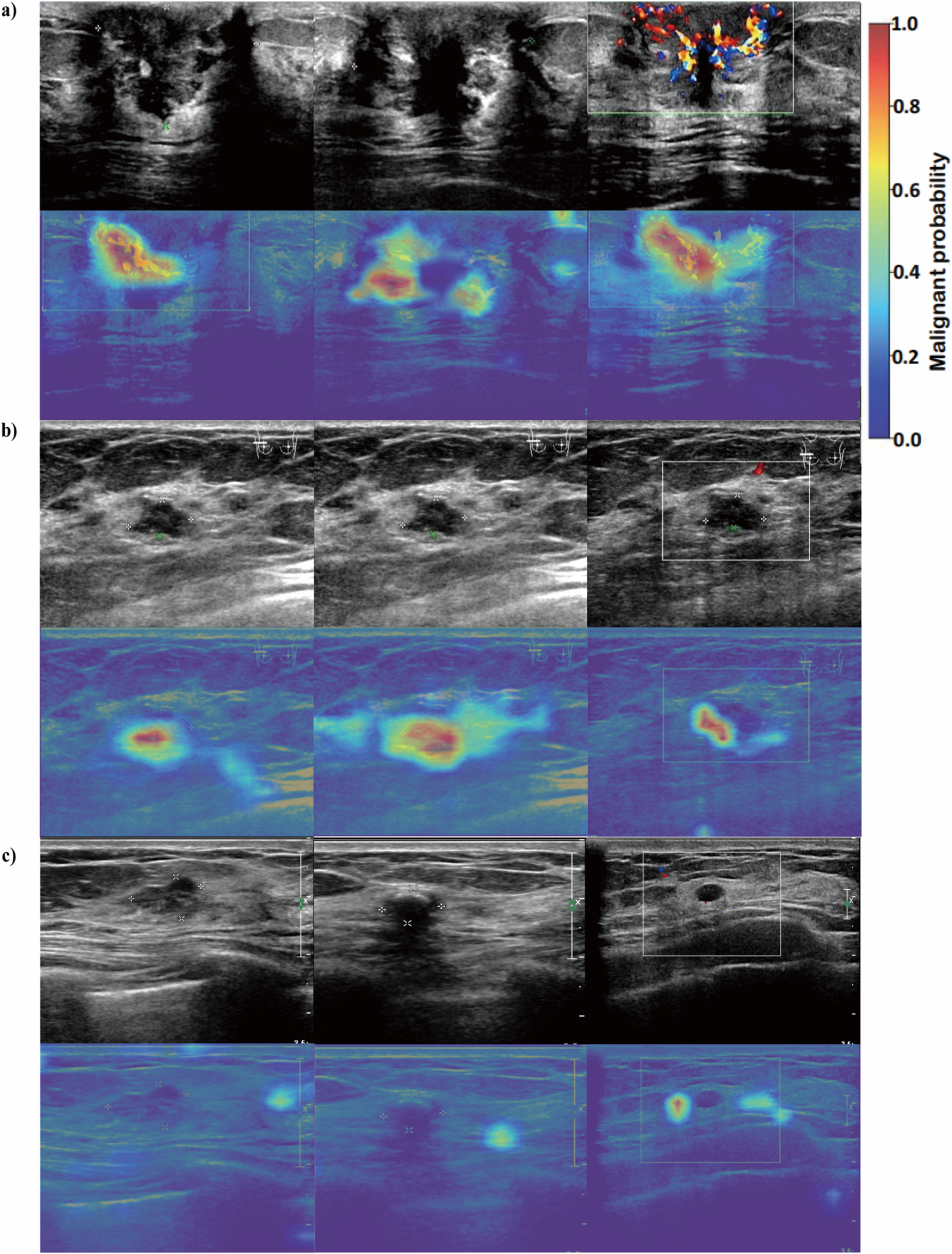
Attention heatmaps visualization of image-based deep learning predictions of malignancy. Visual explanations of DL models are definitely important for qualitative review and clinical relevance, namely, irregular solid components, projections, and areas with abundant blood flow signals. **a** Invasive breast cancer of a 65-year old female; **b** ductal carcinoma in situ of a 68-year old female; **c** breast fibroadenoma of a 55-year old female that was misdiagnosed by all readers but showed a low probability of malignancy in the heatmap. In the first row of each case, the first two images are B-mode images, and the following one is the color Doppler image. The images in the second row are their corresponding heatmaps

Model interpretability results are shown in Figs. 2 and 3, where red regions indicate higher relevance to classification and blue regions indicate lower relevance. In the SJZ dataset, hotspots were identified in 10.32% (352/3409) of benign lesions and 56.54% (255/451) of malignant lesions. In the XT dataset, hotspots were present in 36.15% (539/1491) of benign lesions and 73.24% (52/71) of malignant lesions. These findings suggest clearer and more consistent localization for malignant lesions, which may support interpretability for cancer detection. Feature importance analysis showed that image-based DL predictions contributed most to BrcaDetect decisions, followed by BI-RADS category, family history of breast cancer, age, BMI, and history of benign breast disease (Supplementary Fig. S3).

### Reader study

**Fig. 4:**
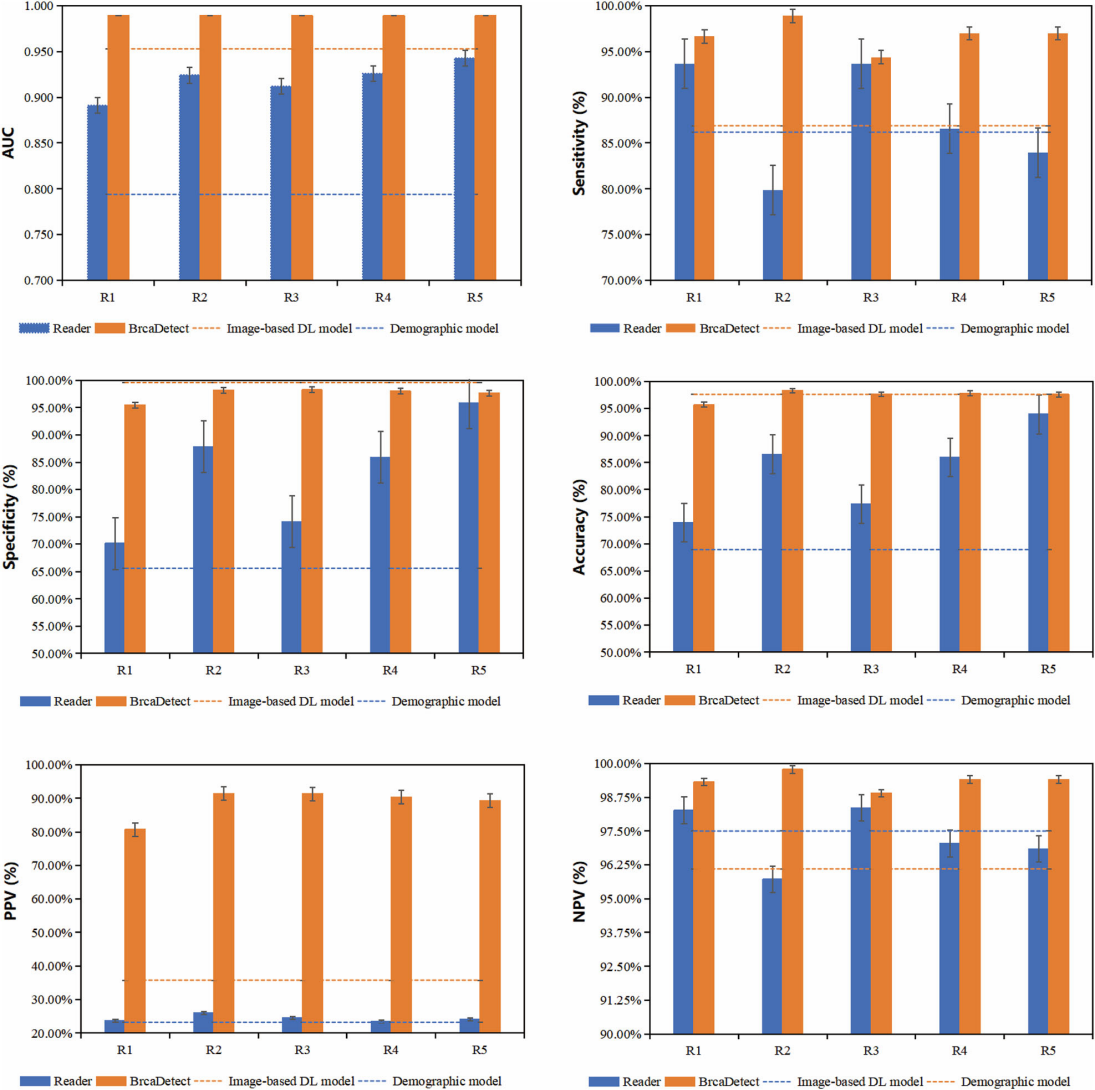
Performance of the image-based deep learning model, demographic model, readers, and the BrcaDetect. We reported the observed values (measure of center) and 95% confidence intervals (error bars) of the AUCs, accuracies, specificities, sensitivities, PPVs, and NPVs of five readers (R1-R5), the image-based deep learning model, the demographic model, and BrcaDetect in the internal test datasets. AUC, area under the receiver operating characteristic curve; PPV, positive predictive value; NPV, negative predictive value

Across all readers, diagnostic performance improved with BrcaDetect assistance. On the internal test dataset, reader AUCs ranged from 0.891 to 0.943. But with BrcaDetect, their AUCs increased, ranging from 0.969 to 0.989 (Fig. 4 and Table 3; *p* < 0.001). Overall, the mean false-positive rate decreased from 17.25% to 2.47% (Table 3), yielding consistent performance gains across readers with different levels of experience. Improvements were most evident among less experienced readers, for whom BrcaDetect was associated with higher diagnostic accuracy and substantially fewer false positives under controlled experimental conditions.

**Table 3:** The diagnostic performance of five radiologists with and without DL assistance

	Type	AUC	*p*-value	Accuracy (%)	Sensitivity (%)	Specificity (%)	PPV (%)	NPV (%)	FPR (%)
Reader 1		0.891	1.015 × 10^−8^	0.739	0.937	0.701	0.379	0.983	29.91%
	+DL	0.970		0.957	0.966	0.955	0.807	0.993	4.51%
Reader 2		0.924	5.729 × 10^−9^	0.865	0.799	0.878	0.562	0.957	12.15%
	+DL	0.989		0.983	0.989	0.982	0.914	0.998	1.82%
Reader 3		0.912	1.93 × 10^−5^	0.773	0.937	0.741	0.414	0.984	25.91%
	+DL	0.969		0.976	0.944	0.983	0.913	0.989	1.74%
Reader 4		0.926	5.908 × 10^−6^	0.860	0.866	0.859	0.545	0.970	14.10%
	+DL	0.980		0.978	0.970	0.980	0.903	0.994	2.00%
Reader 5		0.943	0.0009487	0.924	0.840	0.959	0.798	0.968	4.10%
	+DL	0.980		0.976	0.970	0.977	0.893	0.994	2.30%
Reader average		0.919	2.306 × 10^−5^	0.835	0.877	0.828	0.498	0.972	17.25%
	+DL	0.977		0.974	0.966	0.975	0.884	0.993	2.47%

Inter-reader agreement was evaluated using pairwise Cohen’s kappa coefficients (Supplementary Table S5). For BI-RADS assessments, kappa values ranged from 0.735 to 0.825, and from 0.635 to 0.812 in the internal and external test datasets, indicating fair to excellent agreement. With BrcaDetect assistance, kappa values increased to 0.801–0.890 in the internal test dataset, and 0.760–0.847 in the external dataset, reflecting excellent agreement.

## Discussion

This study suggests that integrating DL‑derived imaging features with radiological assessment and demographic risk factors may assist in image‑level breast cancer risk estimation, rather than providing a standalone patient‑level diagnosis. Across both internal and external test datasets, BrcaDetect consistently outperformed single-modality models, supporting the value of multimodal integration for breast lesion assessment. Comparative analyses further indicated that image-based deep learning predictions provided the strongest discriminative contribution, while BI-RADS assessments and demographic variables added complementary, patient-level information.

Previous studies have demonstrated the feasibility of image-based artificial intelligence for breast lesion classification [25], including multi-center studies such as those by Gu et al [26] and Liao et al [27]. Compared with these image-only approaches, the present study extends existing evidence by showing that incorporating radiological assessment and demographic risk factors yields improved performance within the same datasets. This result suggests that different information sources capture overlapping yet distinct aspects of malignancy risk, and that joint modeling more closely reflects real-world clinical reasoning. Importantly, these conclusions are based on within-study comparisons rather than indirect performance comparisons across cohorts.

Although BrcaDetect demonstrated strong discrimination in the internal test dataset, performance declined in the external datasets, with AUC values ranging from 0.826 to 0.851. This reduction likely reflects domain shift related to differences in ultrasound equipment, acquisition protocols, and population characteristics across centers. Our findings, therefore, serve as proof of concept for image-level assistance, rather than a definitive demonstration of universal clinical utility. These results underscore the need for larger, multi-institutional datasets and the development of domain adaptation strategies to improve generalizability.

The reader study indicated that, under controlled experimental conditions, BrcaDetect was associated with improved diagnostic performance and reduced inter-reader variability, particularly among less experienced radiologists [28]. In contrast, gains were less evident among more experienced readers, who tended to apply more conservative BI-RADS grading, consistent with prior reports [29]. Previous studies have similarly shown that greater experience is associated with more consistent interpretation and fewer false-positive findings [30]. Given the high proportion of benign lesions in the test dataset, the relatively low PPVs observed across all readers are expected and align with real-world screening scenarios.

Several measures were implemented to mitigate anchoring bias during assisted reading, including a 2-week washout period, blinding to prior assessments, and recording BI-RADS categories before disclosure of model output. Only subsequent changes were documented. Although these steps were intended to limit reliance on algorithmic suggestions, residual anchoring effects cannot be entirely excluded. Taken together, the reader study results provide proof-of-concept evidence that BrcaDetect may enhance interpretive consistency and support clinical decision-making under controlled conditions. However, the study was conducted in a simulated environment and did not account for real-world factors such as time pressure, longitudinal clinical context, or downstream management decisions. Accordingly, these findings should be interpreted as proof of concept rather than direct evidence of impact on routine clinical workflows or patient outcomes.

In future clinical practice, BrcaDetect might be considered as a real‑time, image‑level decision‑support tool within ultrasound workflows, pending prospective validation. The model is not designed to replace radiologist judgment or to function as a standalone screening test. Potential applications, currently at the proof‑of‑concept stage, include prioritizing examinations that warrant closer review and serving as a second reader to reduce interobserver variability and reinforce diagnostic confidence. The effectiveness of such integration, however, will require confirmation through prospective evaluation in real-world clinical settings.

Interpretability analyses using Shapley values and Grad‑CAM heatmaps were performed to provide qualitative insights into model behavior. Overall, DL-derived imaging features contributed most strongly to model predictions, followed by BI-RADS assessments and demographic variables. Grad‑CAM visualizations frequently highlighted image regions overlapping with morphologic or vascular patterns commonly regarded as suspicious. These regions should be interpreted as areas of relative model attention rather than precise lesion localization, and interpretability outputs are therefore intended to support understanding of model tendencies rather than to serve as definitive explanatory evidence.

Grad‑CAM heatmaps often emphasized regions with irregular margins, solid components, or increased vascular signals, features previously associated with malignancy in prior studies [31]. However, activation was also observed in some benign lesions, including fibroadenomas and sclerosing adenosis. This overlap likely reflects shared imaging characteristics such as increased vascularity, stromal proliferation, or architectural heterogeneity. Refinement using larger and more diverse benign and normal datasets may help reduce nonspecific activation. Notably, a smaller proportion of malignant lesions showed visually prominent Grad‑CAM hotspots in the external validation cohorts. This observation may be related to differences in acquisition protocols, image quality, transducer settings, lesion characteristics, or patient populations across datasets. These findings highlight the sensitivity of interpretability outputs to dataset characteristics and reinforce the need for cautious interpretation, particularly when models are applied across heterogeneous clinical environments.

This study has several limitations. First, it was retrospective and conducted within a limited geographic region, which may constrain generalizability. Although the evaluation was designed with screening-like conditions, the enriched lesion prevalence and retrospective case selection indicate that it does not represent a population-based screening trial. Instead, it is more appropriately considered a decision-support evaluation in screening-relevant settings. Second, external datasets had relatively small sample sizes and class imbalance, which may affect calibration and performance. Third, heterogeneity in ultrasound devices and acquisition protocols across centers may have contributed to domain shift, and exclusion of low-quality images could introduce selection bias. Future studies should include prospective, multi-center validation in more diverse populations, incorporate additional clinical risk factors, and evaluation across a broader range of image quality and imaging modalities.

## Conclusions

This study supports the potential clinical relevance of the interpretable BrcaDetect system for breast cancer diagnosis. By integrating DL-derived image predictions with demographic information and radiological assessment, the model demonstrated stable performance across both internal and external datasets. These results indicate that multimodal integration may enhance automated breast cancer diagnostic approaches beyond image-only models. Furthermore, the use of Grad‑CAM heatmaps and Shapley value provides insight into the model’s reasoning process and helps delineate the contribution of individual features.

Although BrcaDetect was associated with improved radiologist performance and reduced false-positive rates under controlled experimental conditions, these findings are proof‑of‑concept; further validation in prospective, real‑world settings is required before any clinical implementation. Future studies should explore integration with other imaging modalities, such as mammography and automated breast ultrasound, and conduct prospective, multi-center evaluations to confirm robustness and generalizability across diverse populations.

## Supplementary information

ELECTRONIC SUPPLEMENTARY MATERIAL

## Data Availability

The datasets generated and/or analyzed during the current study are available from the corresponding author on reasonable request.

## Abbreviations

AUC: Area under the curve
BI-RADS: Breast Imaging Reporting and Data System
CIs: Confidence intervals
DL: Deep learning
Grad‑CAM: Gradient-weighted class activation mapping
NPV: Predictive value
PPV: Positive predictive value
RF: Random forest

## References

[1] Bray F, Laversanne M, Sung H et al (2024) Global cancer statistics 2022: GLOBOCAN estimates of incidence and mortality worldwide for 36 cancers in 185 countries. CA Cancer J Clin 74:229–263 38572751 10.3322/caac.21834

[2] Zheng R, Zhang S, Zeng H et al (2022) Cancer incidence and mortality in China, 2016. J Natl Cancer Cent 2:1–9 39035212 10.1016/j.jncc.2022.02.002 PMC11256658

[3] Giaquinto AN, Sung H, Newman LA et al (2024) Breast cancer statistics 2024. CA Cancer J Clin 74:477–495 39352042 10.3322/caac.21863

[4] Zeng H, Zheng R, Sun K et al (2024) Cancer survival statistics in China 2019–2021: a multicenter, population-based study. J Natl Cancer Cent 4:203–213 39281724 10.1016/j.jncc.2024.06.005 PMC11401485

[5] Duggan C, Trapani D, Ilbawi AM et al (2021) National health system characteristics, breast cancer stage at diagnosis, and breast cancer mortality: a population-based analysis. Lancet Oncol 22:1632–1642 34653370 10.1016/S1470-2045(21)00462-9

[6] Hubbard RA, Kerlikowske K, Flowers CI, Yankaskas BC, Zhu W, Miglioretti DL (2011) Cumulative probability of false-positive recall or biopsy recommendation after 10 years of screening mammography: a cohort study. Ann Intern Med 155:481–492 22007042 10.1059/0003-4819-155-8-201110180-00004 PMC3209800

[7] Shen S, Zhou Y, Xu Y et al (2015) A multi-centre randomised trial comparing ultrasound vs mammography for screening breast cancer in high-risk Chinese women. Br J Cancer 112:998–1004 25668012 10.1038/bjc.2015.33 PMC4366890

[8] Lee HJ, Kim EK, Kim MJ et al (2008) Observer variability of Breast Imaging Reporting and Data System (BI-RADS) for breast ultrasound. Eur J Radiol 65:293–298 17531417 10.1016/j.ejrad.2007.04.008

[9] Berg WA, Blume JD, Cormack JB et al (2008) Combined screening with ultrasound and mammography vs mammography alone in women at elevated risk of breast cancer. JAMA 299:2151–2163 18477782 10.1001/jama.299.18.2151 PMC2718688

[10] McKinney SM, Sieniek M, Godbole V et al (2020) International evaluation of an AI system for breast cancer screening. Nature 577:89–94 31894144 10.1038/s41586-019-1799-6

[11] Zhang H, Han L, Chen K, Peng Y, Lin J (2020) Diagnostic efficiency of the breast ultrasound computer-aided prediction model based on convolutional neural network in breast cancer. J Digit Imaging 33:1218–1223 32519253 10.1007/s10278-020-00357-7 PMC7572988

[12] Jiang M, Zhang D, Tang SC et al (2021) Deep learning with convolutional neural network in assessment of breast cancer molecular subtypes based on US images: a multicenter retrospective study. Eur Radiol 31:3673–3682 33226454 10.1007/s00330-020-07544-8

[13] Gu Y, Xu W, Liu T et al (2023) Ultrasound-based deep learning in establishment of a breast lesion risk stratification system: a multicenter study. Eur Radiol 33:2954–2964 36418619 10.1007/s00330-022-09263-8

[14] Hayashida T, Odani E, Kikuchi M et al (2022) Establishment of a deep-learning system to diagnose BI-RADS 4a or higher using breast ultrasound for clinical application. Cancer Sci 113:3528–3534 35880248 10.1111/cas.15511 PMC9530860

[15] Shen Y, Shamout FE, Oliver JR et al (2021) Artificial intelligence system reduces false-positive findings in interpretation of breast ultrasound exams. Nat Commun 12:5645 34561440 10.1038/s41467-021-26023-2 PMC8463596

[16] Edge SB, Compton CC (2010) The American Joint Committee on Cancer: the 7th edition of the AJCC cancer staging manual and the future of TNM. Ann Surg Oncol 17:1471–1474 20180029 10.1245/s10434-010-0985-4

[17] Colditz GA, Atwood KA, Emmons K et al (2000) Harvard report on cancer prevention volume 4: Harvard cancer risk index. Cancer Causes Control 11:477–488 10880030 10.1023/a:1008984432272

[18] Liang G, Zheng L (2020) A transfer learning method with deep residual network for pediatric pneumonia diagnosis. Comput Methods Programs Biomed 187:104964 31262537 10.1016/j.cmpb.2019.06.023

[19] Pachade S, Porwal P, Kokare M, Giancardo L, Mériaudeau F (2021) NENet: Nested Efficientnet and adversarial learning for joint optic disc and cup segmentation. Med Image Anal 74:102253 34614474 10.1016/j.media.2021.102253

[20] Huang G, Liu Z, Pleiss G, Maaten LV, Weinberger KQ (2022) Convolutional networks with dense connectivity. IEEE Trans Pattern Anal Mach Intell 44:8704–8716 31135351 10.1109/TPAMI.2019.2918284

[21] Morid MA, Borjali A, Del Fiol G (2021) A scoping review of transfer learning research on medical image analysis using ImageNet. Comput Biol Med 128:104115 33227578 10.1016/j.compbiomed.2020.104115

[22] Jahmunah V, Ng EYK, Tan RS, Oh SL, Acharya UR (2022) Explainable detection of myocardial infarction using deep learning models with Grad-CAM technique on ECG signals. Comput Biol Med 146:105550 35533457 10.1016/j.compbiomed.2022.105550

[23] Selvaraju RR, Cogswell M, Das A, Vedantam R, Parikh D, Batra D (2017) Grad-CAM: visual explanations from deep networks via gradient-based localization. In: Proceedings of the IEEE international conference on computer vision. IEEE, Venice, pp 618–626

[24] Landis JR, Koch GG (1977) The measurement of observer agreement for categorical data. Biometrics 33:159–174 843571

[25] Łukasiewicz S, Czeczelewski M, Forma A, Baj J, Sitarz R, Stanisławek A (2021) Breast cancer-epidemiology, risk factors, classification, prognostic markers, and current treatment strategies-an updated review. Cancers 13:4287 34503097 10.3390/cancers13174287 PMC8428369

[26] Gu Y, Xu W, Lin B et al (2022) Deep learning based on ultrasound images assists breast lesion diagnosis in China: a multicenter diagnostic study. Insights Imaging 13:124 35900608 10.1186/s13244-022-01259-8 PMC9334487

[27] Liao J, Gui Y, Li Z et al (2023) Artificial intelligence-assisted ultrasound image analysis to discriminate early breast cancer in Chinese population: a retrospective, multicentre, cohort study. EClinicalMedicine 60:102001 37251632 10.1016/j.eclinm.2023.102001 PMC10220307

[28] Xiang H, Xiao Y, Li F et al (2024) Development and validation of an interpretable model integrating multimodal information for improving ovarian cancer diagnosis. Nat Commun 15:2681 38538600 10.1038/s41467-024-46700-2 PMC10973484

[29] Elmore JG, Jackson SL, Abraham L et al (2009) Variability in interpretive performance at screening mammography and radiologists’ characteristics associated with accuracy. Radiology 253:641–651 19864507 10.1148/radiol.2533082308 PMC2786197

[30] Beam CA, Layde PM, Sullivan DC (1996) Variability in the interpretation of screening mammograms by US radiologists: findings from a national sample. Arch Intern Med 156:209–213 8546556

[31] Expert Panel on Breast Imaging, Niell BL, Jochelson MS et al (2024) ACR Appropriateness Criteria® female breast cancer screening: 2023 update. J Am Coll Radiol 21:S126–S143 38823941 10.1016/j.jacr.2024.02.019

