# Supplementary material for: An interpretable ultrasound-based deep learning system for early breast cancer in a Chinese population
Source: Insights Imaging. 2026 Jun 4;17:153. doi: 10.1186/s13244-026-02323-3 (PMC13237303; doi:10.1186/s13244-026-02323-3)

An interpretable ultrasound-based deep learning system for early breast cancer in a Chinese population

ELECTRONIC SUPPLEMENTARY MATERIAL

Table legend

Supplementary Material 1. Detailed information on the risk assessment score.

Supplementary Table S1. Summary of the ultrasound (US) devices.

Supplementary Table S2. Demographic characteristics and associations with breast cancer.

Supplementary Table S3. The performances of different models in participant level.

Supplementary Table S4. The diagnostic performance of radiologists alone, and DL-assisted radiologists.

Supplementary Table S5. Inter-reader agreement (Kappa Coefficients) for diagnostic performance.

Supplementary Material 1. Detailed information on the risk assessment score

Women were defined as being at high risk for breast cancer if they met any of the following criteria:

(1) a positive family history of breast or ovarian cancer;

(2) the presence of any specific high-risk factor, including

a. menarche at age ≤12 years,
b. menopause at age ≥55 years,
c. a history of pathologically confirmed atypical hyperplasia of the breast,
d. having heterogeneously or extremely dense breasts on mammography after age 45; or

(3) at least two moderate-risk factors, including

a. nulliparity or a first live birth at age ≥30 years,
b. no history of breastfeeding or a short breastfeeding duration [<4 months],
c. a history of two or more abortions.

Supplementary Table S1. Summary of the ultrasound (US) devices and linear probes.

Manufacturer	System	Country	Probe settings
Philips	EPIQ7	American	eL18-4
GE	LOGIQ e9	American	ML 6-15
GE	LOGIQ e9	American	9L
TOSHIBA	Aplio 500	Japan	14L5
Siemens	Sequaia	Germany	9L
SAMSUNG	RS80A	Korea	LA2-9A
Hitachi	Aloka	Japan	L441

Supplementary Table S2. **Demographic characteristics and associations with breast cancer.**

	Total (N = 1,732)	Non-breast cancer (N = 1,191)	Breast cancer (N = 541)	*P* value	OR (95%CI)[a]	P values
**Age-group, years**						
40-44	165 (9.5)	115 (9.7)	50 (9.2)	<0.001	*Reference*	
45-49	352 (20.3)	305 (25.6)	47 (8.7)		0.39 (0.25-0.63)	<0.001
50-54	343 (19.8)	260 (21.8)	83 (15.3)		0.83 (0.53-1.30)	0.45
55-59	282 (16.3)	189 (15.9)	93 (17.2)		1.42 (0.91-2.24)	0.103
60-64	339 (19.6)	164 (13.8)	175 (32.3)		3.24 (2.08-5.10)	<0.001
65-69	153 (8.8)	121 (10.2)	32 (5.9)		0.77 (0.45-1.33)	0.36
70-74	98 (5.7)	37 (3.1)	61 (11.3)		4.70 (2.69-8.33)	<0.001
**BMI (kg/m^2)**						
<18.5	7 (0.4)	7 (0.6)	0 (0.0)	<0.001	-	0.966
18.5-23.9	850 (49.1)	598 (50.2)	252 (46.6)		*Reference*	
24.0-27.9	656 (37.9)	466 (39.1)	190 (35.1)		0.97 (0.75-1.23)	0.776
⩾28.0	219 (12.6)	120 (10.1)	99 (18.3)		1.92 (1.37-2.69)	<0.001
**Education level**				0.08		

Low	185 (10.7)	132 (11.1)	53 (9.8)		*Reference*	
Medium	1175 (67.8)	788 (66.2)	387 (71.5)		1.31 (0.89-1.91)	0.174
High	372 (21.5)	271 (22.8)	101 (18.7)		1.13 (0.73-1.76)	0.593
**Smoking status**						
Never	1616 (93.3)	1111 (93.3)	505 (93.3)	0.736	*Reference*	
Current	98 (5.7)	69 (5.8)	29 (5.4)		0.76 (0.41-1.38)	0.379
Ever	18 (1.0)	11 (0.9)	7 (1.3)		1.31 (0.43-3.79)	0.625
**Alcohol consumption**						
Never	1600 (92.4)	1111 (93.3)	489 (90.4)	0.102	*Reference*	
Current	111 (6.4)	68 (5.7)	43 (7.9)		1.45 (0.84-2.49)	0.177
Ever	21 (1.2)	12 (1.0)	9 (1.7)		1.33 (0.49-3.48)	0.563
**Age of menarche (y)**						
<13	279 (16.1)	205 (17.2)	74 (13.7)	0.074	0.78 (0.57-1.07)	0.134
⩾13	1453 (83.9)	986 (82.8)	467 (86.3)		*Reference*	
**Menopausal status**						
Premenopausal	685 (39.5)	484 (40.6)	201 (37.2)	0.186	*Reference*	
Postmenopausal	1047 (60.5)	707 (59.4)	340 (62.8)		0.76 (0.58-1.00)	0.052
**Delivery history**						

No	76 (4.4)	46 (3.9)	30 (5.5)	0.145	*Reference*	
Yes	1656 (95.6)	1145 (96.1)	511 (94.5)		0.70 (0.40-1.25)	0.222
**Breastfeeding**						
No	178 (10.3)	119 (10.0)	59 (10.9)	0.62	*Reference*	
Yes	1554 (89.7)	1072 (90.0)	482 (89.1)		0.95 (0.65-1.40)	0.791
**Personal history of breast benign disease**						
No	1259 (72.7)	927 (77.8)	332 (61.4)	<0.001	*Reference*	
Yes	473 (27.3)	264 (22.2)	209 (38.6)		1.55 (1.00-2.43)	0.053
**Family history of breast cancer**						
No	1134 (65.5)	843 (70.8)	291 (53.8)	<0.001	*Reference*	
Yes	598 (34.5)	348 (29.2)	250 (46.2)		1.54 (1.00-2.35)	0.046

*Abbreviation: BMI, Body mass index,

a. ORs were adjusted for factors including age, BMI, benign breast disease and family history of breast cancer in the multivariate logistic regression model.

Supplementary Table S3. The performances of different models in participant level

Internal test dataset	Densenet121	Densenet169	Densenet201	Efficientnet_b5	Efficientnet_b6	Resnet34	Resnet50	Resnet101
AUC	0.947	0.952	0.954	0.949	0.942	0.941	0.962	0.957
Sensitivity (%)	0.810	0.806	0.799	0.825	0.836	0.806	0.825	0.825
Specificity (%)	1.000	1.000	1.000	1.000	0.998	1.000	1.000	1.000
Accuracy (%)	0.969	0.968	0.967	0.971	0.971	0.968	0.971	0.971
PPV (%)	1.000	1.000	1.000	1.000	0.987	1.000	1.000	1.000
NPV (%)	0.964	0.964	0.962	0.967	0.969	0.964	0.967	0.967
**SJZ test dataset**								
AUC	0.790	0.729	0.710	0.705	0.766	0.752	0.810	0.788
Sensitivity (%)	0.412	0.594	0.441	0.652	0.517	0.727	0.663	0.576
Specificity (%)	0.953	0.790	0.895	0.607	0.881	0.668	0.827	0.876
Accuracy (%)	0.890	0.767	0.842	0.612	0.839	0.675	0.808	0.841
PPV (%)	0.538	0.272	0.358	0.180	0.365	0.225	0.336	0.380
NPV (%)	0.925	0.936	0.924	0.930	0.932	0.949	0.949	0.940
**XT test dataset**								
AUC	0.744	0.790	0.813	0.626	0.752	0.845	0.735	0.748
Sensitivity (%)	0.718	0.606	0.479	0.775	0.915	0.394	0.958	0.620
Specificity (%)	0.622	0.808	0.907	0.284	0.448	0.943	0.339	0.686
Accuracy (%)	0.627	0.799	0.888	0.307	0.469	0.918	0.367	0.683
PPV (%)	0.083	0.131	0.198	0.049	0.073	0.248	0.065	0.086
NPV (%)	0.979	0.977	0.973	0.964	0.991	0.970	0.994	0.974

Supplementary Table S4. The diagnostic performance of radiologists alone, and DL-assisted radiologists.

	Reader 1		Reader 2		Reader 3		Reader 4		Reader 5		Average	
**Internal set**	BI-RADS	BrcaDetect	BI-RADS	BrcaDetect	BI-RADS	BrcaDetect	BI-RADS	BrcaDetect	BI-RADS	BrcaDetect	BI-RADS	BrcaDe[illegible]
AUC	0.891	0.970	0.924	0.989	0.912	0.969	0.926	0.980	0.943	0.980	0.919	0.97[illegible]
SEN (%)	0.937	0.966	0.799	0.989	0.937	0.944	0.866	0.970	0.840	0.970	0.875	0.96[illegible]
SPE (%)	0.701	0.955	0.878	0.982	0.741	0.983	0.859	0.980	0.959	0.977	0.828	0.97[illegible]
ACC (%)	0.739	0.957	0.865	0.983	0.773	0.976	0.860	0.978	0.939	0.976	0.835	0.97[illegible]
PPV (%)	0.379	0.807	0.562	0.914	0.414	0.913	0.545	0.903	0.798	0.893	0.539	0.88[illegible]
NPV (%)	0.983	0.993	0.957	0.998	0.984	0.989	0.970	0.994	0.968	0.994	0.972	0.99[illegible]
FPV (%)	0.299	0.045	0.122	0.018	0.259	0.017	0.141	0.020	0.041	0.023	0.172	0.02[illegible]
**SJZ dataset**												
AUC	0.678	0.826	0.803	0.831	0.750	0.843	0.745	0.822	0.779	0.829	0.751	0.83[illegible]
SEN (%)	0.654	0.718	0.776	0.681	0.716	0.701	0.676	0.718	0.701	0.725	0.705	0.70[illegible]
SPE (%)	0.722	0.870	0.708	0.918	0.708	0.918	0.708	0.868	0.708	0.869	0.711	0.88[illegible]
ACC (%)	0.714	0.852	0.716	0.891	0.709	0.893	0.705	0.851	0.708	0.852	0.710	0.86[illegible]
PPV (%)	0.237	0.422	0.260	0.525	0.245	0.532	0.235	0.419	0.241	0.422	0.244	0.46[illegible]
NPV (%)	0.940	0.959	0.960	0.956	0.950	0.959	0.943	0.959	0.947	0.960	0.948	0.95[illegible]
FPV (%)	0.278	0.130	0.292	0.082	0.292	0.082	0.292	0.132	0.292	0.131	0.289	0.11[illegible]
**XT dataset**												
AUC	0.660	0.768	0.715	0.791	0.645	0.792	0.661	0.776	0.671	0.798	0.670	0.78[illegible]
SEN (%)	0.507	0.873	0.69	0.873	0.493	0.873	0.493	0.873	0.577	0.873	0.552	0.87[illegible]
SPE (%)	0.762	0.714	0.691	0.73	0.756	0.767	0.746	0.717	0.721	0.745	0.735	0.73[illegible]
ACC (%)	0.751	0.722	0.691	0.737	0.744	0.771	0.735	0.724	0.714	0.751	0.727	0.74[illegible]
PPV (%)	0.092	0.127	0.096	0.134	0.088	0.151	0.085	0.128	0.09	0.14	0.090	0.13[illegible]
NPV (%)	0.97	0.992	0.979	0.992	0.969	0.992	0.969	0.992	0.973	0.992	0.972	0.99[illegible]
FPV (%)	0.237	0.286	0.309	0.270	0.244	0.233	0.254	0.283	0.279	0.255	0.265	0.26[illegible]

Supplementary Table S5. Inter-reader agreement (Kappa Coefficients) for diagnostic performance

		Readers	Internal test dataset	SJZ External test dataset	XT External test dataset
Inter-reader	BI-RADS	R1 vs R2	0.774(0.750-0.796)	0.749(0.734-0.764)	0.730(0.685-0.777)
		R1 vs R3	0.750(0.725-0.774)	0.684(0.665-0.702)	0.704(0.659-0.747)
		R1 vs R4	0.750(0.725-0.775)	0.738(0.721-0.755)	0.751(0.700-0.803)
		R1 vs R5	0.751(0.726-0.775)	0.761(0.745-0.777)	0.772(0.732-0.814)
		R2 vs R3	0.777(0.753-0.803)	0.717(0.700-0.732)	0.728(0.687-0.770)
		R2 vs R4	0.738(0.712-0.765)	0.777(0.761-0.793)	0.635(0.585-0.687)
		R2 vs R5	0.825(0.802-0.847)	0.794(0.778-0.809)	0.812(0.773-0.844)
		R3 vs R4	0.743(0.715-0.768)	0.711(0.694-0.728)	0.559(0.503-0.612)
		R3 vs R5	0.763(0.737-0.789)	0.729(0.710-0.747)	0.741(0.701-0.778)
		R4 vs R5	0.735(0.708-0.761)	0.771(0.754-0.786)	0.759(0.710-0.804)
	BrcaDetect	R1 vs R2	0.824(0.800-0.848)	0.814(0.801-0.829)	0.736(0.690-0.780)
		R1 vs R3	0.801(0.777-0.824)	0.760(0.743-0.776)	0.780(0.737-0.821)
		R1 vs R4	0.816(0.791-0.841)	0.809(0.794-0.824)	0.757(0.709-0.803)
		R1 vs R5	0.828(0.805-0.850)	0.825(0.810-0.839)	0.786(0.743-0.826)
		R2 vs R3	0.817(0.793-0.840)	0.782(0.767-0.799)	0.782(0.741-0.818)
		R2 vs R4	0.849(0.825-0.871)	0.838(0.825-0.852)	0.638(0.588-0.690)
		R2 vs R5	0.878(0.858-0.898)	0.847(0.834-0.861)	0.812(0.772-0.850)
		R3 vs R4	0.890(0.869-0.909)	0.783(0.766-0.798)	0.629(0.578-0.679)
		R3 vs R5	0.833(0.810-0.856)	0.790(0.773-0.805)	0.801(0.762-0.838)
		R4 vs R5	0.853(0.830-0.875)	0.830(0.816-0.844)	0.759(0.709-0.806)

Data in parentheses are 95% confidence intervals; BI-RADS - Breast Imaging Reporting and Data System

Supplementary Fig. S1 Flow chart of patients' selection. To employ the ultrasound (US) image datasets from two medical centres, our deep learning system was developed and internally validated based on US image.

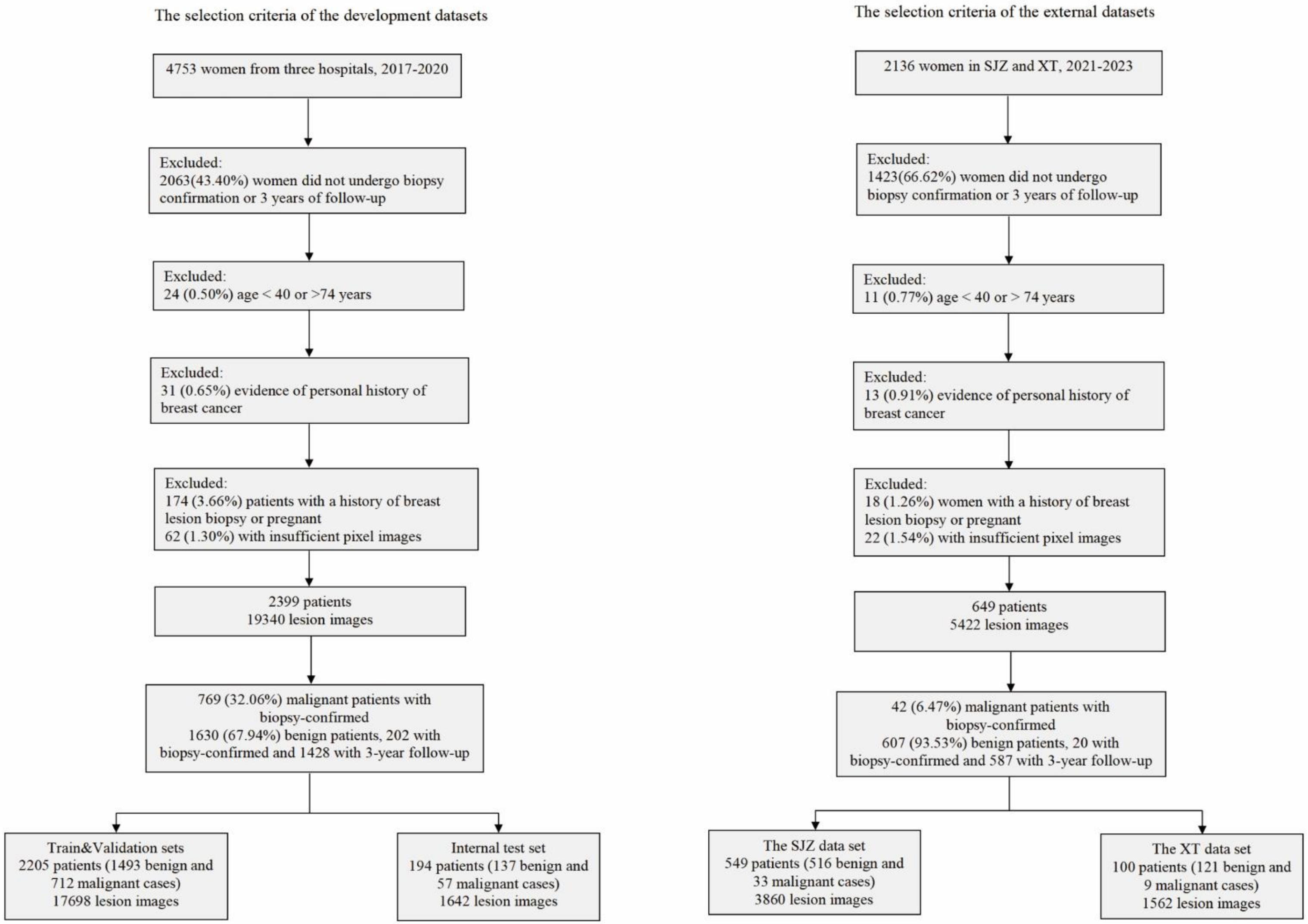
The selection criteria of the development datasets
4753 women from three hospitals, 2017-2020
Excluded:
2063(43.40%) women did not undergo biopsy confirmation or 3 years of follow-up
Excluded:
24 (0.50%) age < 40 or >74 years
Excluded:
31 (0.65%) evidence of personal history of breast cancer
Excluded:
174 (3.66%) patients with a history of breast lesion biopsy or pregnant
62 (1.30%) with insufficient pixel images
2399 patients
19340 lesion images
769 (32.06%) malignant patients with biopsy-confirmed
1630 (67.94%) benign patients, 202 with biopsy-confirmed and 1428 with 3-year follow-up
Train&Validation sets
2205 patients (1493 benign and 712 malignant cases)
17698 lesion images
Internal test set
194 patients (137 benign and 57 malignant cases)
1642 lesion images
The selection criteria of the external datasets
2136 women in SJZ and XT, 2021-2023
Excluded:
1423(66.62%) women did not undergo biopsy confirmation or 3 years of follow-up
Excluded:
11 (0.77%) age < 40 or > 74 years
Excluded:
13 (0.91%) evidence of personal history of breast cancer
Excluded:
18 (1.26%) women with a history of breast lesion biopsy or pregnant
22 (1.54%) with insufficient pixel images
649 patients
5422 lesion images
42 (6.47%) malignant patients with biopsy-confirmed
607 (93.53%) benign patients, 20 with biopsy-confirmed and 587 with 3-year follow-up
The SJZ data set
549 patients (516 benign and 33 malignant cases)
3860 lesion images
The XT data set
100 patients (121 benign and 9 malignant cases)
1562 lesion images

Supplementary Fig. S2 Receiver operator characteristic curves of the three models. (a) Internal test dataset; (b) SJZ external test dataset; and (c) XT external test dataset.

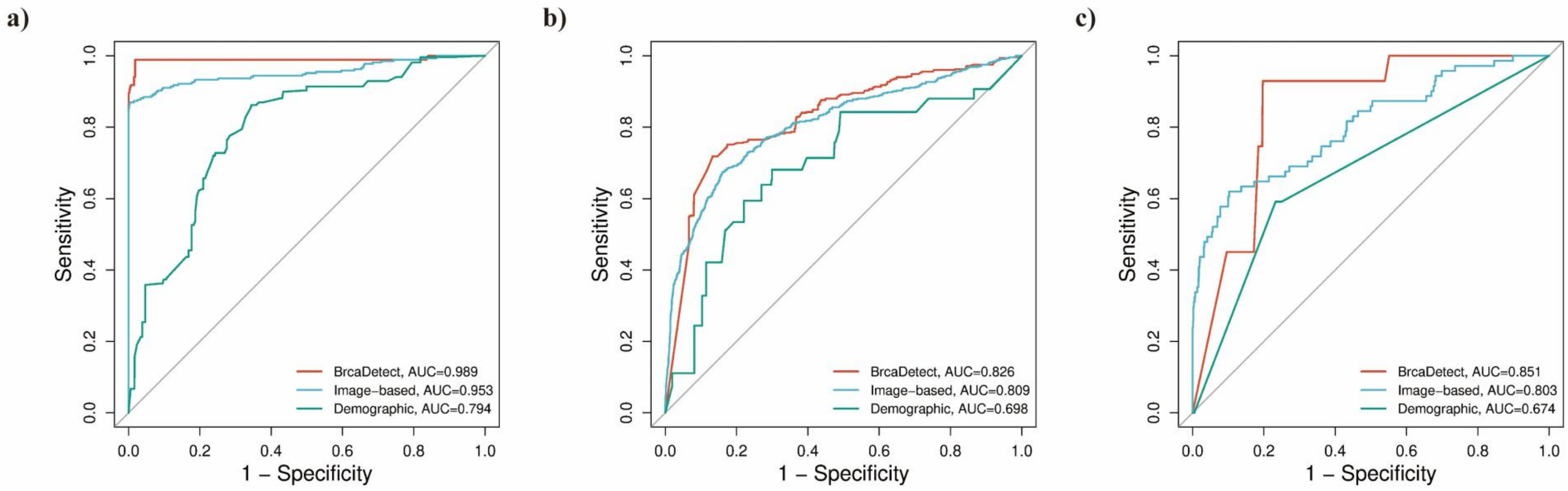

Supplementary Fig. S3 Global Shapley values for the interpretation of BrcaDetect. The horizontal ordinate represents the mean absolute Shapley value, indicating the global importance weights of the features. Each dot represents an individual patient. The higher the Shapley values, the greater probability of breast cancer.

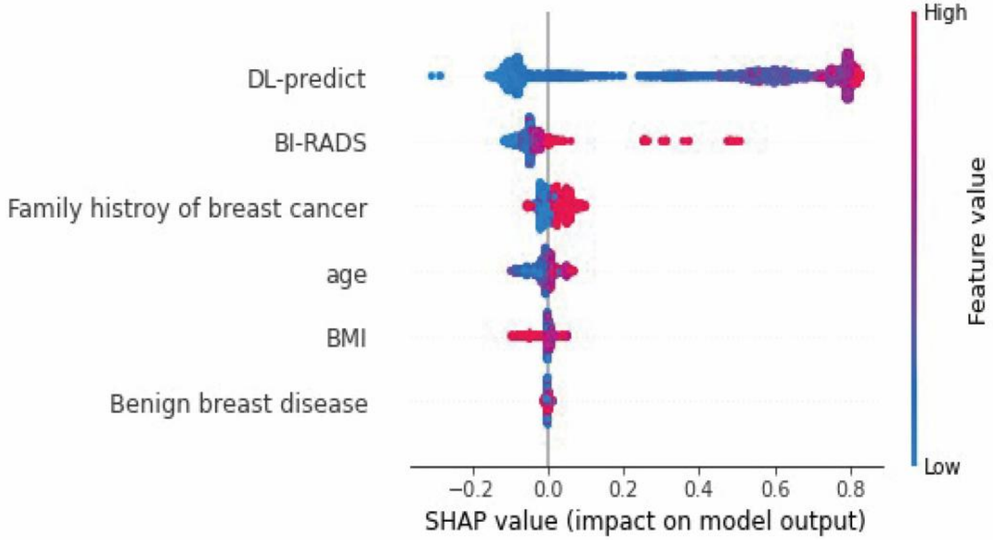

Supplement: Supplementary file 1 — ELECTRONIC SUPPLEMENTARY MATERIAL [file 13244_2026_2323_MOESM1_ESM.pdf]
